# Supplementary material for: Structural basis of human PCNA sliding on DNA
Source: Nat Commun. 2017 Jan 10;8:13935. doi: 10.1038/ncomms13935 (PMC5234079; doi:10.1038/ncomms13935)
Supplement: Supplementary Information — Supplementary Figures, Supplementary Tables and Supplementary References [file ncomms13935-s1.pdf]

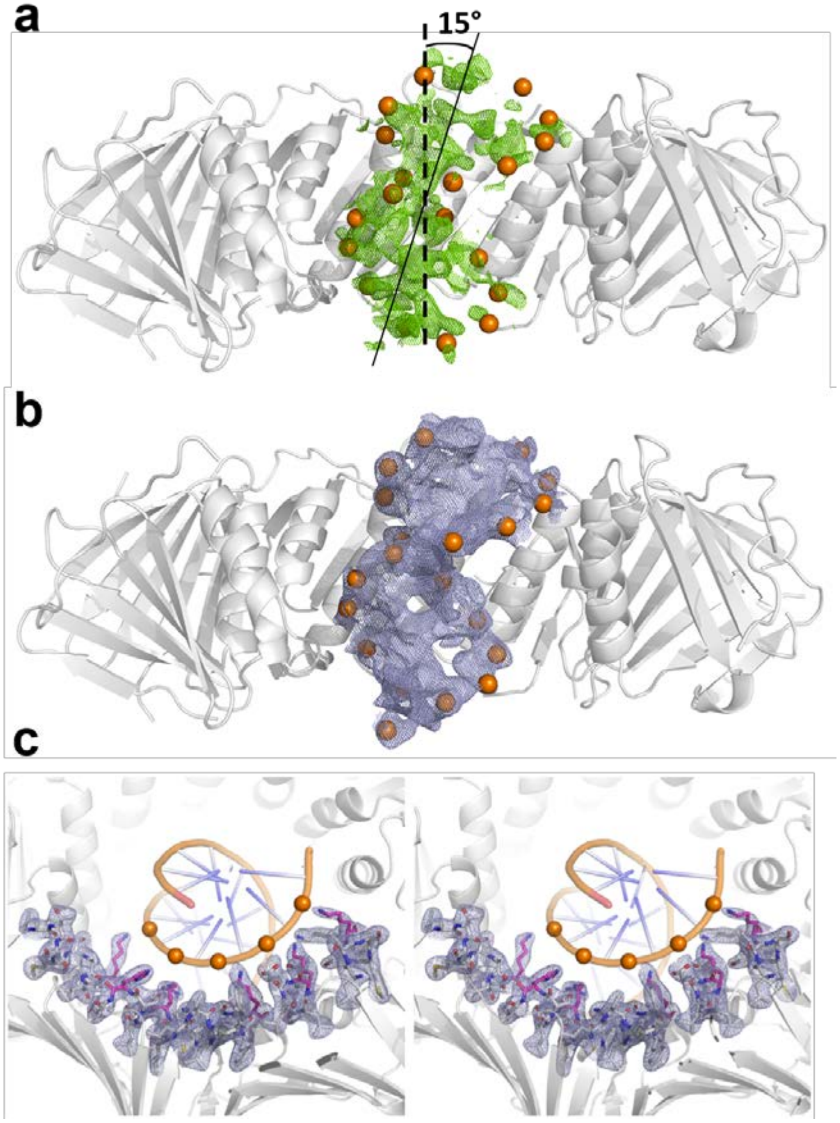

Supplementary Figure 1. (**a**) Side view of the Fo-Fc map of the PCNA–dsDNA complex before adding DNA to the model, contoured at 1.3 σ. DNA is tilted 15° from the C3 rotation axis of PCNA (**b**) Side view of the final 2Fo-Fc map, contoured at 0.8 σ. The positions of DNA phosphates in the final model are indicated as orange spheres. The PCNA subunit in the foreground is not shown for clarity (**c**) Stereo view of the 2Fo-Fc map contoured at 1.3 σ. The figure shows the quality of the 2.82 Å map for the PCNA residues contacting DNA (purple sticks) and their neighbor residues (white sticks).

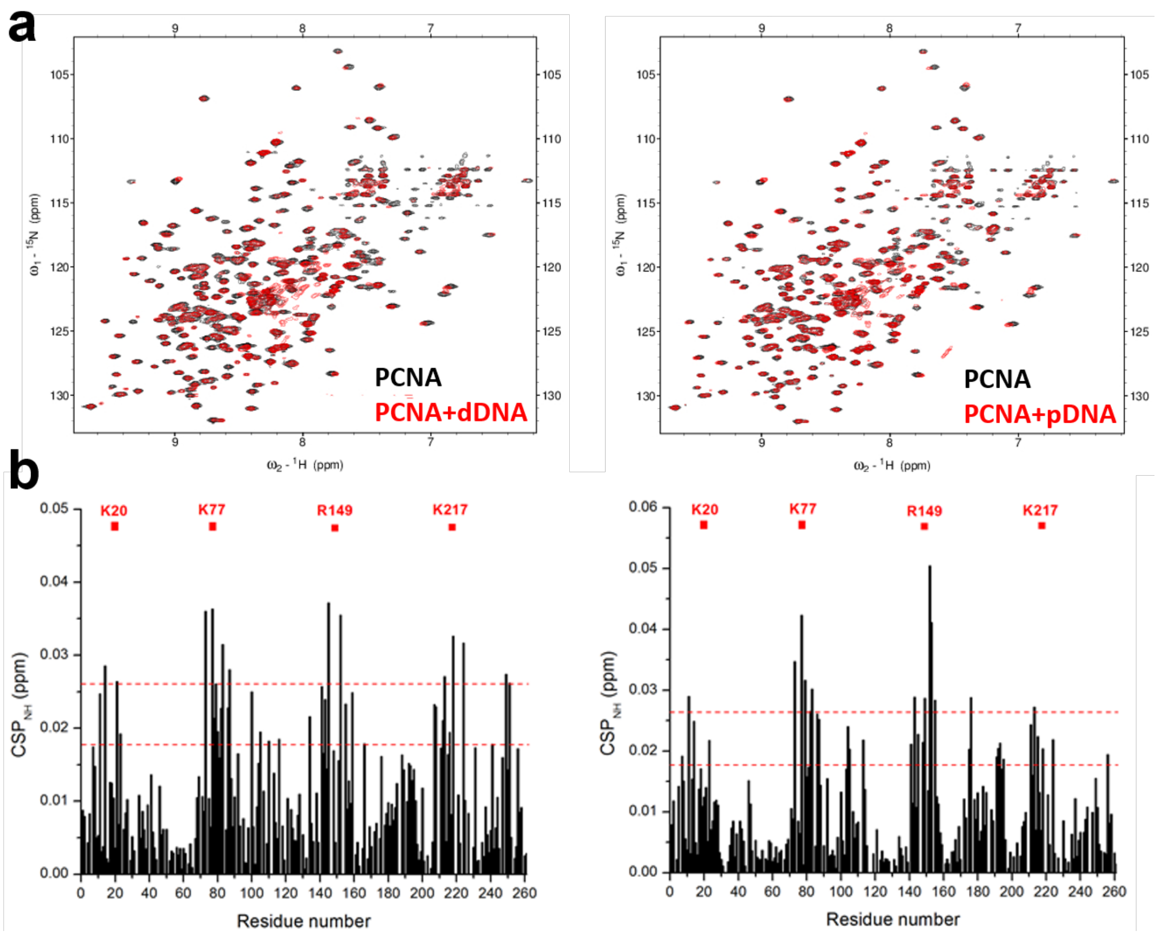

Supplementary Figure 2. *NMR analysis of PCNA binding to dsDNA or pDNA* (**a**) Superposition of ^{1}H-^{15}N TROSY spectra of 100 μM PCNA in the absence (black) and presence (red) of 1.3 mM dsDNA (left) or 1.6 mM pDNA (right), generated with oligonucleotides 1-3 in Supplementary Table 1. Spectra were acquired at 35 °C on samples in 20 mM sodium phosphate, 50 mM NaCl, pH 7.0 (**b**) Chemical shift perturbations (CSP) of backbone amide ^{1}H and ^{15}N NMR resonances induced by (left) dsDNA or (right) pDNA. The dotted lines indicate the average plus one or two standard deviations. The assignment coverage of non-proline PCNA residues bound to dsDNA or pDNA was 90%. The positions of the four positively charged residues interacting with consecutive phosphates in the PCNA–dsDNA crystal structure are indicated above the bars.

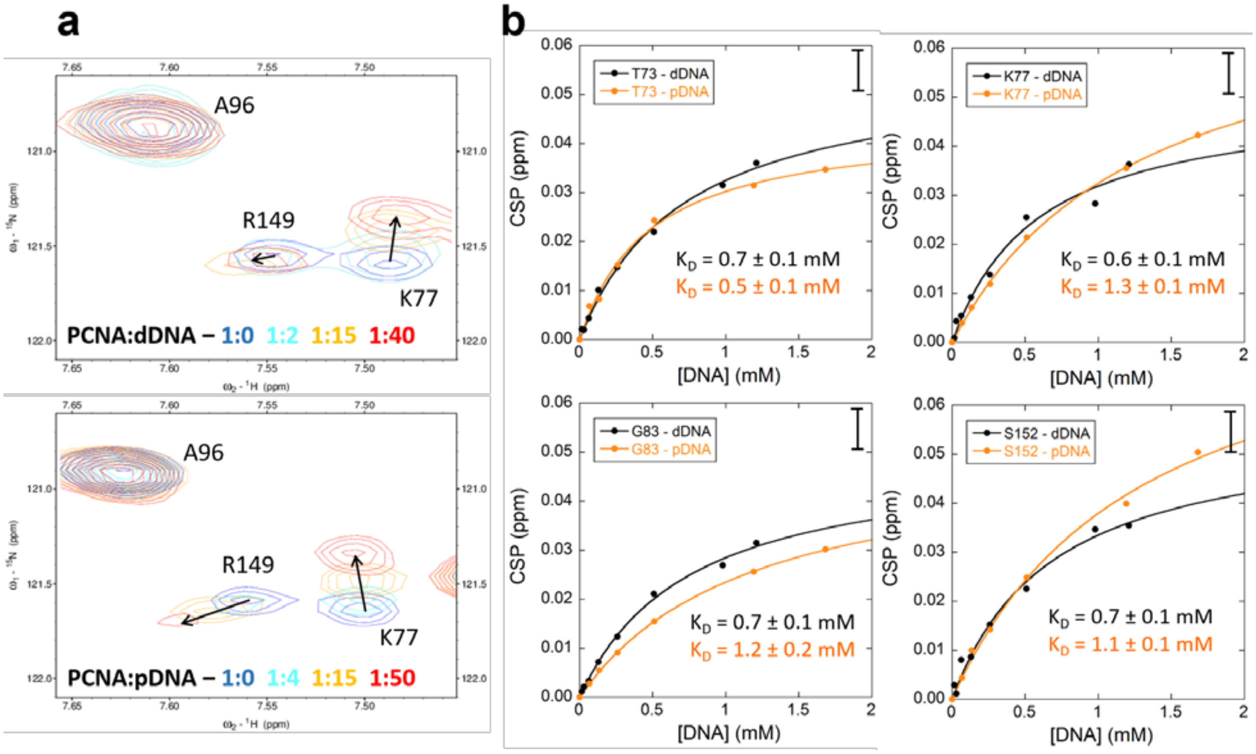

Supplementary Figure 3. *NMR titration analysis of PCNA binding to dsDNA or pDNA.* (**a**) Overlay of ^{1}H-^{15}N TROSY spectra of 100 µM PCNA in the presence of increasing concentrations of unlabeled dsDNA or pDNA, as indicated by the PCNA trimer:DNA duplex molar ratios. For the sake of clarity only four points along the titration are plotted. Spectra were measured in 20 mM phosphate buffer, 50 mM NaCl, pH 7.0, at 35 ºC. The selected region shows significantly perturbed residues (K77, R149) and an unperturbed residue (A96). Arrows indicate the peak center of the residue at the last titration point. The steady shifts of the signals imply a fast exchange between free and bound PCNA with respect to the NMR chemical shift time scale (**b**) Analysis of NMR CSP of selected PCNA residues at increasing concentrations of (black) dsDNA or (orange) pDNA, using a single-site binding model. The errors in the K_D values are fitting errors. The CSP experimental error estimated from the digital resolution of the spectra is 0.009 ppm, which is the size of the bar at the right upper corner of the plots. This is, however, a

conservative estimation since for most residues in regions far away from the inner side of the ring CSP < 0.005 ppm (Fig. S2). For dsDNA, the extrapolated perturbation of a single resonance frequency (^{15}N of K77) at saturation is 0.32 ± 0.03 ppm (or 26 ± 2 Hz). This value, and the fast exchange regime, indicates that the lifetime of the complex is much shorter than 38 ms.

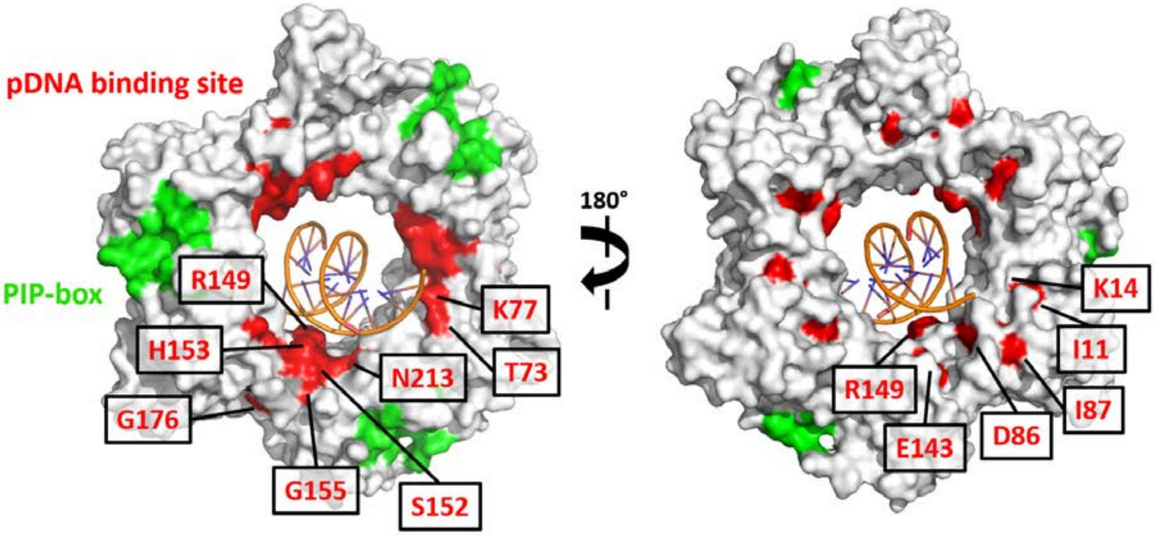

Supplementary Figure 4. *NMR mapping of the pDNA binding site on the PCNA surface* (left, front face; right, back face). PCNA residues whose NMR signals are significantly perturbed by pDNA (CSP > average + 2 standard deviations) are colored red and labeled. Residues belonging to the PIP-box binding site are colored green. DNA is shown in the PCNA–dsDNA complex crystallographic position.

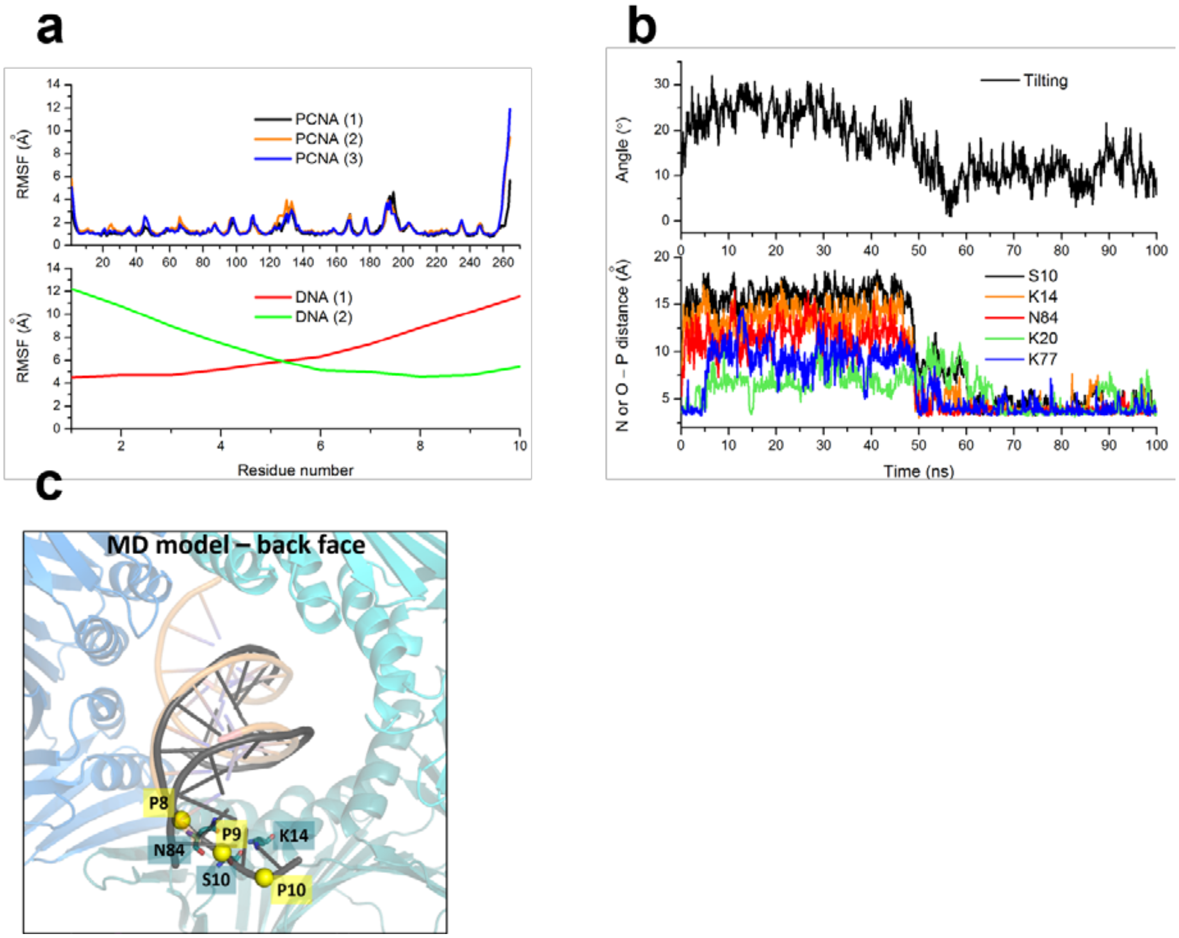

Supplementary Figure 5. *Analysis of the MD simulation of PCNA bound to the 10 bp DNA duplex* (**a**) Root-mean-square-fluctuation (RMSF) of the PCNA Cα atoms and the DNA C1' atoms in the MD trajectory. The three PCNA subunits and two DNA strands are numbered (**b**) *Upper panel*: Evolution of DNA tilting relative to the ring plane. *Lower panel*: Evolution of protein side chain nitrogen (N) or oxygen (O) to DNA phosphorus (P) distances in the MD trajectory for the indicated PCNA residues, which are all located on a single subunit (**c**) MD interactions at the clamp back face. The crystallographic position of the DNA segment is shown in orange, whereas in black is shown DNA in a position corresponding to the final state of the MD simulation. Interacting DNA phosphates and PCNA side chains are shown as yellow spheres and sticks, respectively.

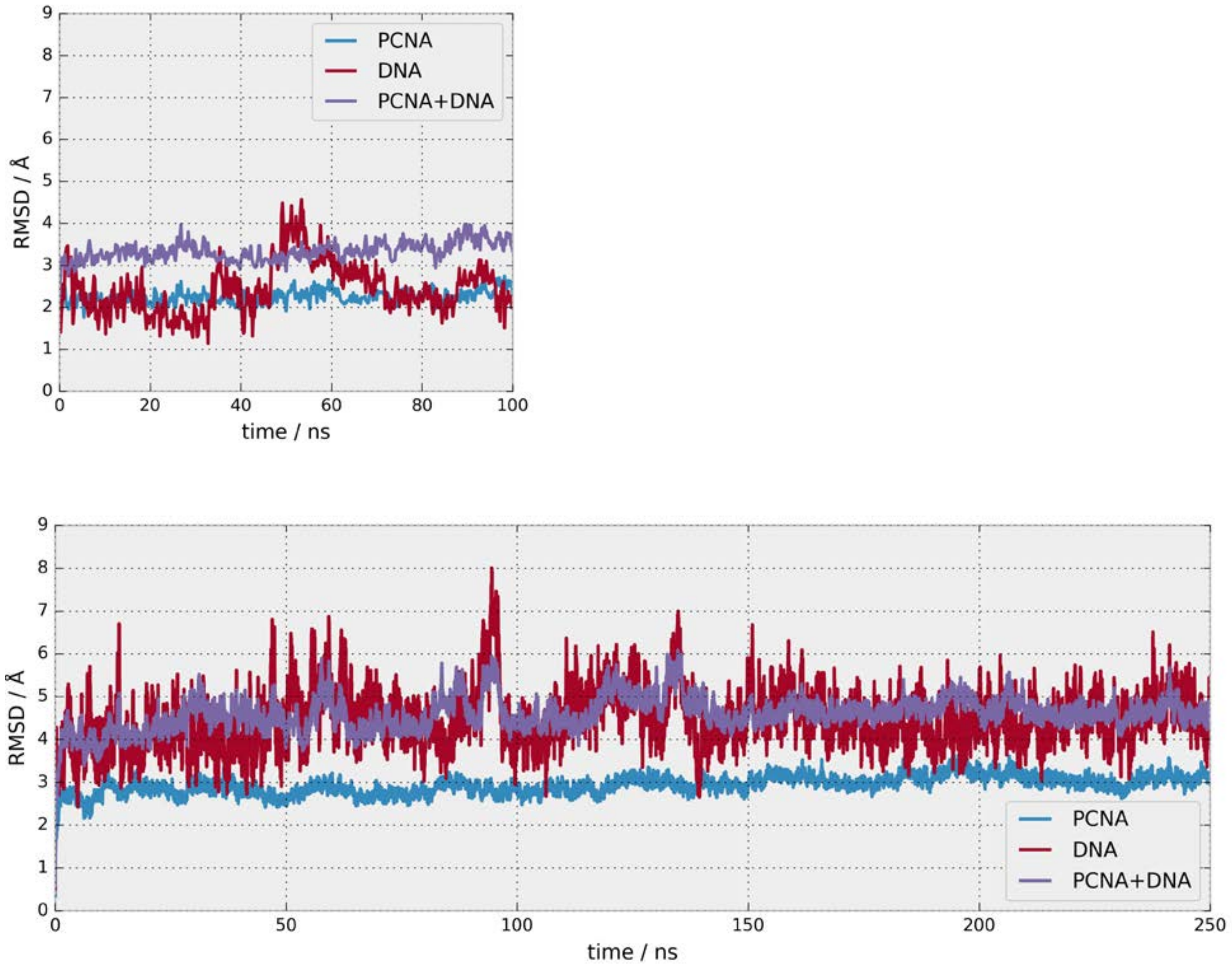

Supplementary Figure 6. Root Mean Square Deviation (RMSD) calculated for the backbone heavy atoms of the PCNA protein, the DNA duplexes, and complexes along the MD trajectories. In each case, the system has been superimposed onto the initial minimized structure that was built from the crystal structure as explained in the methods section. *Upper panel*: 100 ns simulation of PCNA in complex with the 10 bp DNA. *Lower panel*: 250 ns simulation of PCNA in complex with the 30 bp DNA. The spike in RMSD of DNA at around 90 ns arises from a local wagging motion of the 30 bp DNA ends. For the sake of comparison, the same vertical and horizontal scales have been used.

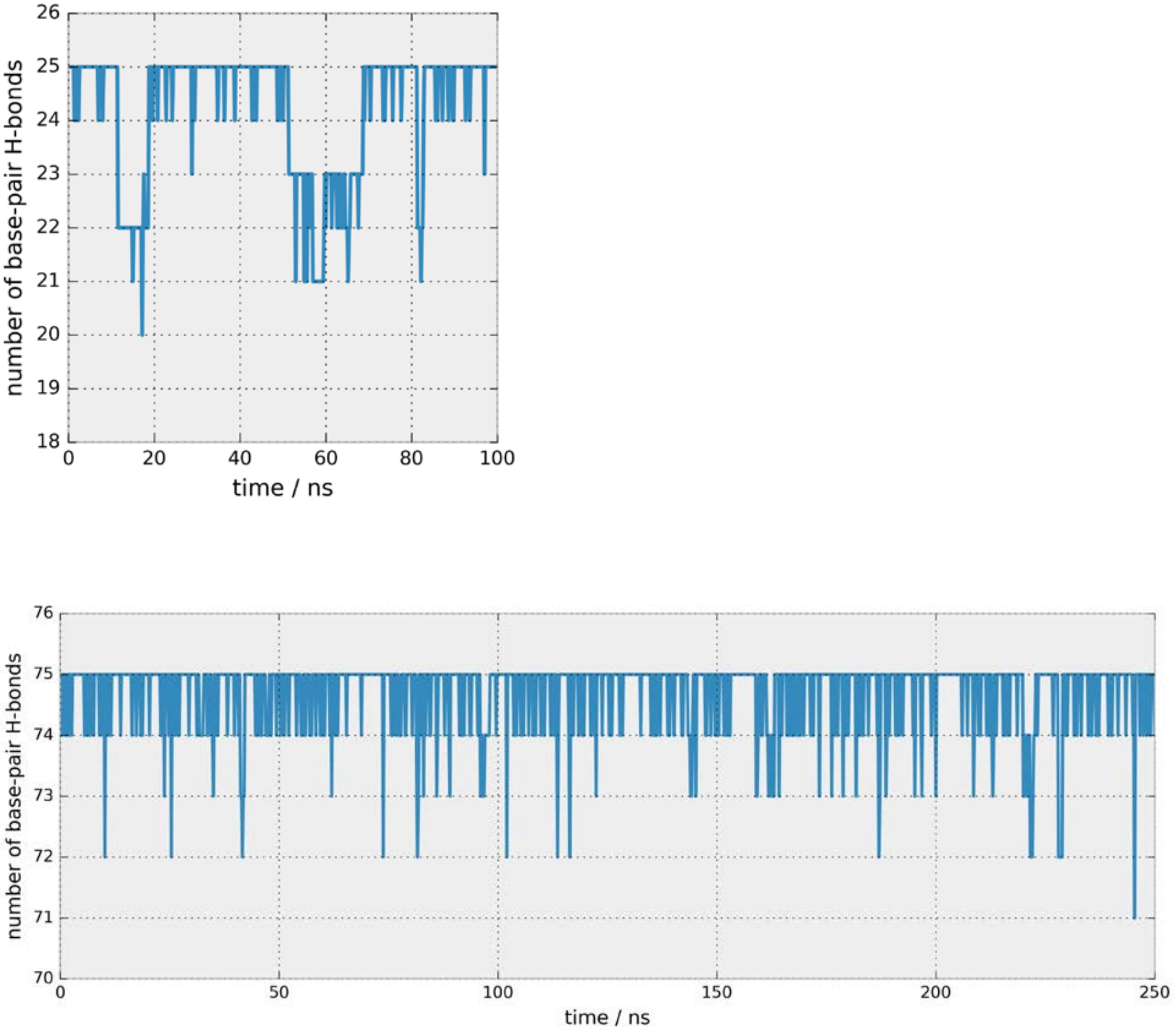

Supplementary Figure 7. *Number of H-bonds between DNA base pairs*. *Upper panel*: 10 bp DNA in the 100 ns trajectory. *Lower panel*: 30 bp DNA in the 250 ns trajectory. The figure shows that the DNA remains in its double stranded form during all the trajectory.

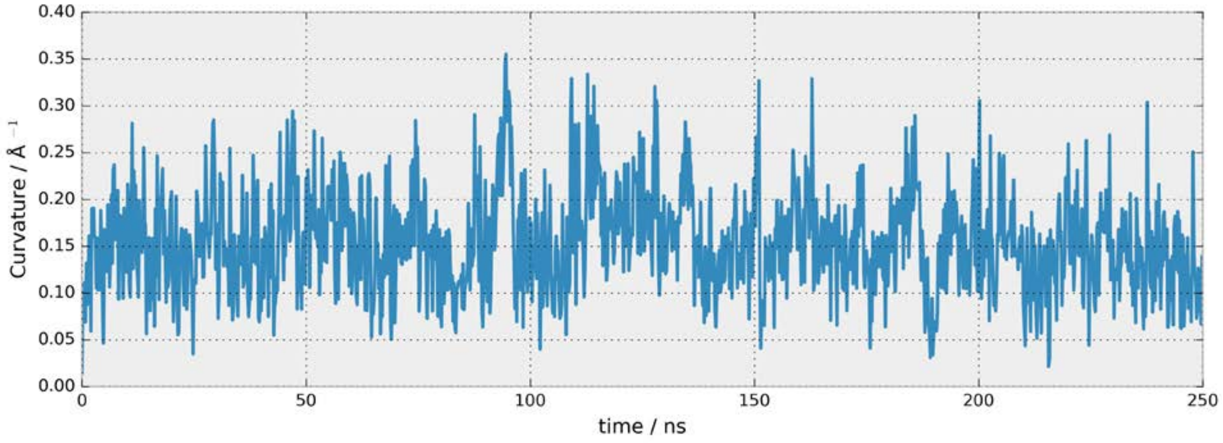

Supplementary Figure 8. *Cumulative DNA curvature*. Curvature calculated from base pair 5 to 25 for the simulation relative to the 30 bp DNA. The figure shows that the DNA has little curvature and that the trajectory is stable. Curvature of the 10 bp DNA was not calculated because the DNA segment is too short for such an analysis[1].

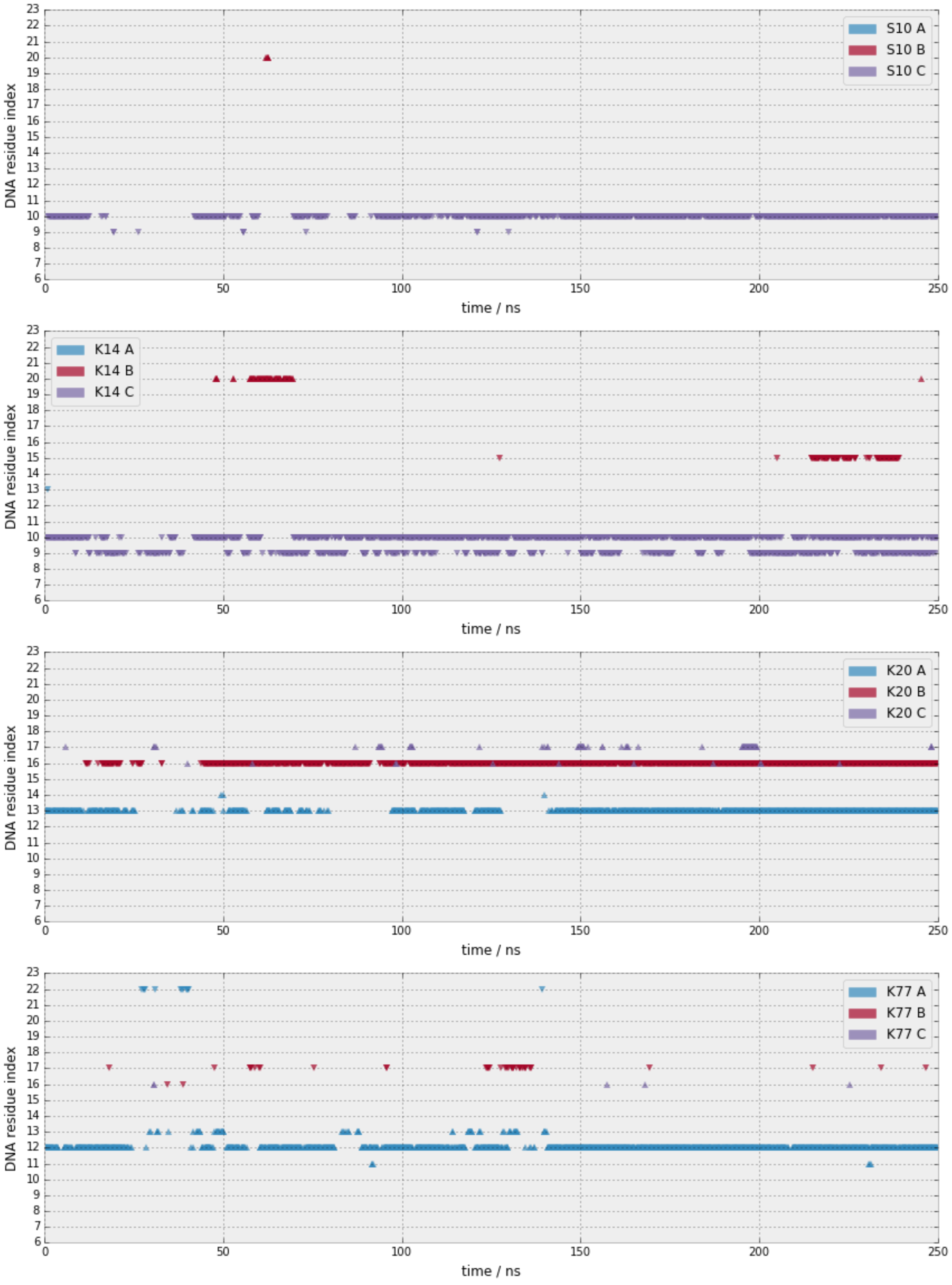

DNA residue index
time / ns
S10 A
S10 B
S10 C
K14 A
K14 B
K14 C
K20 A
K20 B
K20 C
K77 A
K77 B
K77 C

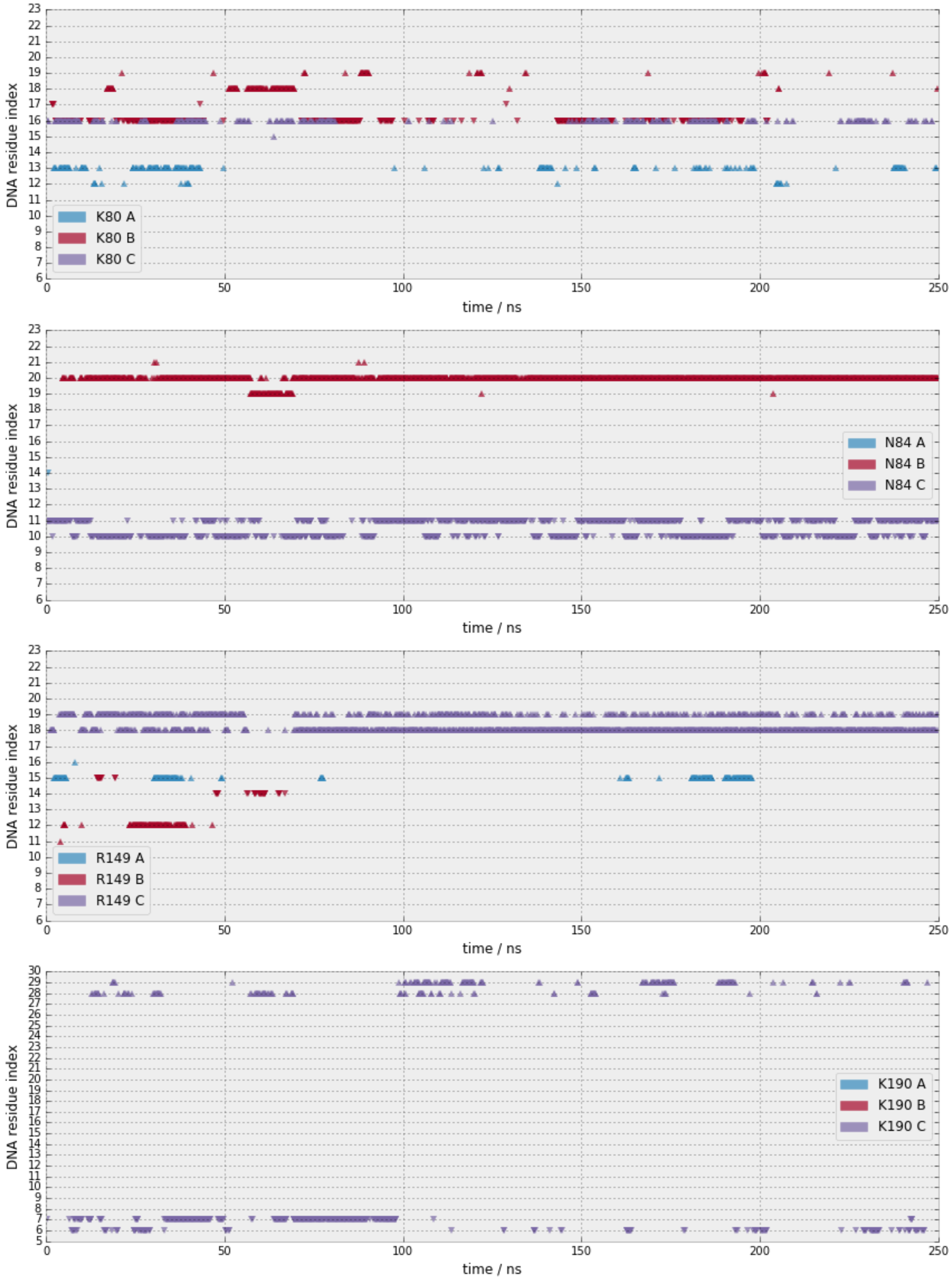
DNA residue index
time / ns
K80 A
K80 B
K80 C
N84 A
N84 B
N84 C
R149 A
R149 B
R149 C
K190 A
K190 B
K190 C

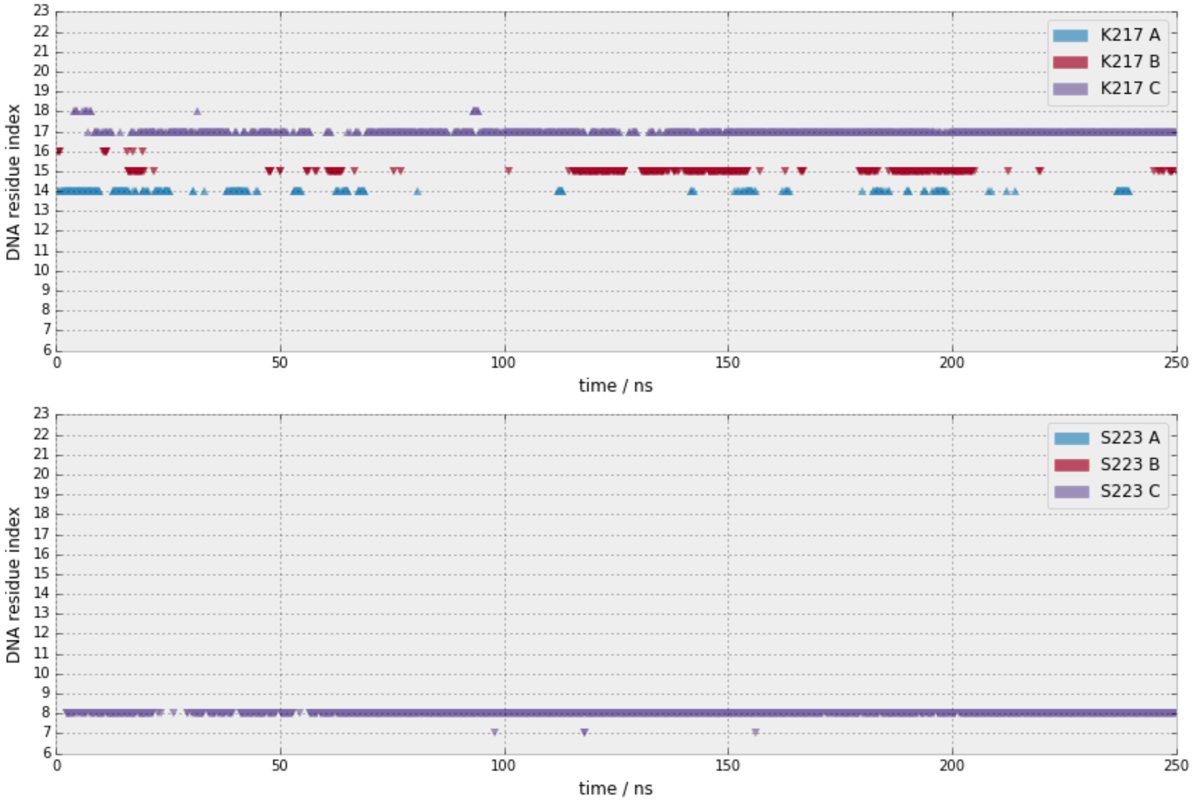

Supplementary Figure 9. *Analysis of the MD simulation of PCNA bound to the 30 bp DNA duplex*. Time evolution of contacts (interatomic side chain nitrogen or oxygen – DNA phosphorus distance < 4.7 Å) between PCNA interfacial residues and DNA phosphorus atoms. Contacts with residues belonging to PCNA subunit A, B and C are shown as triangles colored in turquoise, red and purple, respectively. The nucleotides of the two strands of the 30 bp dsDNA are consecutively numbered from 1 to 20, and triangles with vertexes pointing up and down correspond to contacts with DNA strand 1 and 2, respectively.

Supplementary Table 1. Data collection and refinement statistics (molecular replacement)

	PCNA–dsDNA (PDB: 5L7C)
**Data collection**	
Space group	H3
Cell dimensions	
a, *b*, *c* (Å)	180.19, 180.19, 76.83
α, β, γ (°)	90, 90, 120
Resolution (Å)	90.1 - 2.82 (2.97 - 2.82)
R_{merge}	0.093 (0.44)
$I / \sigma I$	2.7 (1.7)
Completeness (%)	99.6 (99.8)
Redundancy	3.1 (3.0)
**Refinement**	
Resolution (Å)	90.09 - 2.82
No. reflections	21184
R_{work} / R_{free}	0.250 / 0.285
No. atoms	
Protein	5349
Ligand/ion	410
Water	31
B-factors (Å^2)	
Protein	44.25
Ligand/ion	193.67
Water	59.25
R.m.s. deviations	
Bond lengths (Å)	0.0206
Bond angles (°)	2.2194

Supplementary Table 2. Sequences of the oligonucleotides used in this study

DNA oligo	Sequence (5´-3´)
1	ATACGATGGG
2	CCCATCGTAT
3	TTTTATACGATGGG
4	ATACGATGGGATACGATGGGATACGATGGG
5	CCCATCGTATCCCATCGTATCCCATCGTAT

The sequence of oligonucleotides 1 and 2 were used to form the 10 bp dsDNA. The sequences of oligonucleotides 2 and 3 were used to form the primed (p) DNA and correspond to the substrate used in the crystal structure of β-clamp bound to pDNA, reported by Georgescu *et al.*[2]. Oligonucleotides 4 and 5 were used to form the 30 bp DNA for the corresponding MD simulation.

Supplementary References

1. Goodsell, D.S. & Dickerson, R.E. Bending and curvature calculations in B-DNA. *Nucleic Acid Res.* **22**, 5497–5503 (1994)

2. Georgescu, R. E. *et al*. Structure of a sliding clamp on DNA. *Cell* **132**, 43-54 (2008)
